# A report of the in-farm variation of antimicrobial use in commercial broiler production in Pakistan using an international monitoring system based on treatment frequency

**DOI:** 10.3389/fvets.2025.1650299

**Published:** 2025-11-06

**Authors:** Betty Rehberg, Sandra Brogden, Fariha Fatima, Muhammad Umair, Maria Hartmann, Julia Kschonek, Umar Farooq, Mashkoor Mohsin, Lothar Kreienbrock

**Affiliations:** 1 Institute of Biometry, Epidemiology and Information Processing, University of Veterinary Medicine Hannover, Hannover, Germany; 2 Institute of Microbiology, University of Agriculture, Faisalabad, Pakistan; 3 Department of Biology, INEOS Oxford Institute for Antimicrobial Research, University of Oxford, Oxford, United Kingdom; 4 NS Poultry, Faisalabad, Pakistan

**Keywords:** poultry, critically important antibiotics, low- and middle-income countries, farm level monitoring, Asia

## Abstract

Reducing antimicrobial use (AMU) in animal husbandry is imperative to curb the rising threat of antimicrobial resistance. Therefore, sustainable monitoring of AMU is essential to ensure responsible use, minimize resistance and promote long-term effectiveness. Examining the on-farm AMU in broiler production in Pakistan aimed to encourage farmers to adopt responsible antimicrobial practices, while also helping to observe trends in AMU during the fattening period as well as differences between farms. The data were obtained using the international AMU monitoring system VetCAb-ID (©TiHo Hannover, Germany). In this study, the results of monitoring four commercial broiler farms, each with 20 flocks, were investigated for a period of one year. Treatment frequency (TF) based on Used Daily Dose was used to determine flock, farm and season specific differences in AMU. Describing the relative TF of different antimicrobial classes. Shows that the use of antimicrobial classes varied between farms, among flocks within a farm and across fattening weeks within a flock. Overall, the most frequently used classes were polymyxins (27.2%), fluoroquinolones (20.4%), macrolides (17.1%) and tetracyclines (15.9%). The TF was higher in winter than in summer flocks. A statistically significant difference between summer and winter flocks could be observed in the use of fluoroquinolones (*p* = 0.0463) and macrolides (*p* = 0.0325). Using the shared international database VetCAb-ID, detailed and internationally comparable information on the on-farm use of antibiotics in Pakistan broiler production could be obtained and analyzed to identify differences between farms and flocks.

## 1 Introduction

Antimicrobial resistance (AMR) has been recognized as a major global health threat. Reduction of antimicrobial use (AMU) in human and veterinary medicine is one of the central pillars of global and subsequent national action plans on AMR (1). Low and middle-income countries bear the highest burden of drug-resistant infections (2).

Recently, compared to previous reports the World Organization for Animal Health recorded an overall global increase in AMU in animals of 2% (3). Moreover, it has been suggested that global antimicrobial sales will increase by 11.5% until 2030 (4). A recent study suggests that AMU in livestock could rise to 143,481 tons globally by 2040 (5). Monitoring AMU is the key factor for the development of antimicrobial stewardship programs and comparison between farms, regions and countries.

However, monitoring of veterinary AMU by using a harmonized system across sectors remains challenging. Currently, no harmonized monitoring system exists for the collection of AMU data worldwide (6). In Europe, many countries annually report the antimicrobial use in food animals to the European Medicines Agency’s (EMA) European Surveillance for Veterinary Antimicrobial Consumption based on sales data using treatment incidence including European Defined Daily Doses (DDD) and Population Correction Unit as units of measurement (7). Sales data is usually based on several assumptions and does not consider the animal species, number of animals treated, treatments, and dosing differences. Therefore, the EMA suggests farm-level AMU monitoring, which provides more accurate usage data. In addition, it is needed to reduce irrational use, to improve animal husbandry and disease prevention and to support benchmarking purposes.

The Pakistani poultry sector is one of the leading sectors of country’s Gross Domestic Product and is ranked 11^th^ among the world’s largest poultry producers (8). Due to increasing demand of poultry-based food, farmers use large quantities of antimicrobials to control diseases. However, AMR in poultry is a growing challenge in Pakistan and has been attributed to excessive use of antimicrobials (9, 10). In addition, antimicrobial growth promoters are also used in the poultry sector in Pakistan on a routine basis (11).

There is no consensus on the use of AMU metrics in food animals. The choice depends on the purpose of surveillance as discussed by Sanders et al. (12). To introduce two common metrics, used daily dose (UDD) accounts for the actual used dose of an antimicrobial active substance per kg animal body weight per day. DDD is defined as the average maintenance dose per day for a drug which is often recorded from the Summary of Product Characteristics. A recent study showed significant deviation between the DDD for Pakistani poultry and dosing standards established by the EMA. This difference highlights the need for country specific dosing standards to quantify AMU (13). Furthermore, Kasabova et al. (14) could show that the discrepancies between the assumed standard weight used in DDD calculation and the actual animal weight at the time of the treatment used in UDD calculation can lead to large differences in AMU calculation in poultry and suggest using UDD and the UDD based treatment frequency (TF) for the quantification of farm-level AMU. AMU data from Pakistani poultry farms was previously documented using the shared international database VetCAb-ID (15). Building on those past experiences, the database was now used to collect data from four farms in the Punjab region over the course of one year to describe differences between flocks and farms in overall AMU and the use of antibiotic classes. Additionally, flock size, duration of the fattening period and season were taking into account as possibly influencing factors.

The estimation of AMU in food animals is essential to devise and implement antimicrobial stewardship programs in the veterinary sector. The systematic collection and analysis of use data for antimicrobials in animals can provide valuable insights into veterinary prescribing practices and work toward optimizing AMU, improve animal health and combat the AMR. This study is particularly significant because it provides valuable insight into antibiotic consumption in a region, where documentation is not mandatory and therefore limited data has previously been available. The standardised documentation system used enables direct international comparison of the data, as well as detailed analysis of variability in antibiotic consumption at individual farm level. These findings are essential for identifying differences between production sites or management units, and for developing targeted measures to optimise antibiotic use. Thus, the study makes a significant contribution to strengthening global efforts in the fight against AMR and to the promotion of sustainable strategies in veterinary medicine.

## 2 Method

A closed cohort from four controlled-house-type commercial broiler farms in Faisalabad, Pakistan consisting of 20 flocks (five flocks per farm) was investigated for a period of one year (January 2022–December 2022) to collect AMU data and to determine the flock by flock and the farm by farm variation of antimicrobial usage. The selected farms routinely maintained records of medicines used in their farm registers as part of standard commercial broiler production practices in Punjab. To minimize recall bias in this study, a field veterinarian and a researcher from the University of Agriculture in Faisalabad visited the farms weekly to review and verify the records. Both the field veterinarian and the researcher received standardized training on data collection procedures prior to this study.

The prospective study was conducted using the international AMU monitoring system VetCAb-ID (“Veterinary Consumption of Antibiotics-International Documentation”). The system, originally developed in Germany, is a global database that serves as a web-based infrastructure for users in the veterinary field with the primary objective to facilitate the monitoring, tracking, and analysis of AMU data (15). To use the database, VetCAb-ID manuals as well as videos are available for educating data collectors. Videos are available on the homepage of the data base. A pre-structuring tool with information on all the data needed for the VetCAb-ID database, which can be used offline (Excel sheet), is also available for project partners and data editors. Detailed experiences with the use of this database in Pakistan poultry farms were previously published (15).

To facilitate AMU data collection on farm-level in Pakistan, specific (paper based) data collection form was designed and provided to farm veterinarians in the national language. The form was used to document comprehensive details about the correspondent (data collector) and the broiler farm such as the name of the correspondent, farm ID, date of the flock placement, average days of the production cycle, flock size reared, average mortality per flock, average final weight per bird, tentative diagnosis/prophylactic, the name, brand and amount of antimicrobial drug used, route of application, and duration of the treatment. Data were collected on-site prospectively and included all antimicrobial treatments during the complete fattening period of each flock, starting with the placing of day-old chicks at the farms. All documented antimicrobials were prescribed by a certified veterinarian.

The collected, paper based AMU data and active ingredients calculations were first documented with the program Microsoft excel 365. The data was later transferred to the VetCAb-ID database for standardization. The database included data entry control mechanisms such as warnings when the number of animals treated exceeds the number of day-old chicks placed at the farm to avoid some input errors. The data were then initially checked for completeness, plausibility and inconsistencies such as unusual duration of the fattening period, number of animals, or treatment duration using SAS 9.4 M7 (SAS Institute Inc., Cary, NC, United States). All inconsistencies could be corrected by consulting the prescribing veterinarian. The data were then prepared for further descriptive and statistical analysis.

To determine the flock-by-flock and the farm-by-farm variation of antimicrobial usage in broiler farms in Pakistan, the TF per flock was used.


$$\mathrm{TF}=\frac{\sum_{\text{treatments}}(\mathrm{No}.\text{of animals treated}\times \mathrm{No}.\text{of treatment days})}{\mathrm{No}.\text{of animals in the population}}$$

The TF is a count-based quantification of the AMU and directly indicates the on-farm use. First the number of single treatments (one animal treated with one active ingredient for one day) was calculated by multiplying the number of treated animals with the number of treatment days and the number of active ingredients for each treatment. Secondly, the sum of single treatments for each flock was calculated and then divided by the number of animals in the flock. The TF represents the average number of days a broiler has been treated over the time of the fattening period of the flock, e.g., average used daily doses (UDDs) administered to one animal for a particular duration (14). The TF was also calculated for each antimicrobial class and fattening week by assigning the single treatments to respective antimicrobial classes/fattening weeks and only considering t from that class when calculating the TF for each flock. The relative TF for each antimicrobial class and fattening week was then calculated by dividing its TF by the total TF.

Median and mean TF values, inter quartile ranges and standard deviations were used to describe the TF, relative TF values are presented as percentages. The differences between farms and flocks were further described using information about the indications of the treatments, total TF as well as TF and relative TF of the different antimicrobial classes and fattening weeks. To calculate the TF for different seasons, the summer months were subdivided into second half mid-March to first half mid-September and the winter months into second half mid-September to first half mid-March. The seasons were broadly divided into summer and winter, because some flocks were placed in mid-spring, but experienced hot summer months by the end of their production cycle (e.g., April, when temperatures exceed 40 °C) and other flocks were placed in September but completed production in November. The timing of flock placement was determined by the farm owners based on their convenience and was not influenced by the researchers. All farms were environmentally controlled houses with detailed records of temperature (26 to 33 °C) and humidity (70 to 75%).

SAS 9.4 M7 (SAS Institute Inc., Cary, NC, United States) was used for the calculation of the TF, descriptive tables and figures as well as Wilcoxon two-sample tests for non-parametric one-factorial group comparisons of overall TF and TF for each antimicrobial class to compare antibiotic use in regard to season, flock size and duration of the fattening period. *p*-values are presented as results of the non-parametric tests.

Due to the limited samples size of 20 flocks, multi-factorial group comparisons were not possible (there were too few observations per subgroup), and the corresponding models could not be calculated.

## 3 Results

### 3.1 Sample population under study

Four broiler farms with 20 flocks (5 flocks per farm) were included in this study over an observation period of one year. The length of the production cycle for each flock ranged from 26 to 58 days with an average of 40 days. The number of day-old chicks placed per flock ranged from 24,000 to 67,000, and the total number of broilers studied for all farms over the one-year period was 860,900. The majority of flocks (15 of 20) started in the first half of the year between January and June. Only five flocks started between July and November. Following the definition of summer and winter stated in the method section, nine flocks started in summer and 11 flocks in winter.

### 3.2 Inter- and intra-farm variation of the TF

In total, 126 antimicrobial treatments with 20 different drugs containing 1 to 5 active ingredients were recorded. No flock without antimicrobial use was recorded. Per flock, 4 to 9 treatments occurred with an average of 6.3 treatments. Indications include growth promotion (16 treatments) and prophylaxis (1 treatment) as well as respiratory tract (2 treatments), *E. coli* (29 treatments), *Mycoplasma* (54 treatments) and *Salmonella* (24 treatments) infections. One farm used growth promotion treatments in all 5 flocks, two farms in 4 of 5 flocks and one farm used growth promotion in 3 of 5 flocks. All flocks have been treated for *Mycoplasma* and *E. coli; Salmonella* treatments occurred in 2 to 5 flocks per farm.

The sum of TF in all flocks was 576.5 in 782 fattening days. Table 1 shows the TF of each flock: The TF per flock ranged from 14 to 61. The median TF for all flocks was 27 and ranged from 23 to 32 for each farm. The two smaller farms 113 and 116 (between 24,000 and 30,600 animals per flock) show lower median TFs and inter quartile ranges (IQR) than the two larger farms 114 and 115 (between 52,000 and 67,000 animals per flock). The flocks with a shorter fattening period (less than 38 days) had a lower median TF (22.5) compared to the flocks with a longer fattening period (30.5). The median and mean TF was lower in summer (median = 24, mean = 26.5) than in winter (median = 29, mean = 31.7) flocks (Supplementary material 1).

**Table 1:** Information about the treatment frequency (TF), the number of day-old chicks placed (flock size), duration of the fattening period in days (fattening period) and season for each flock (1 to 5) of each farm (113, 114, 115, 116): information on treatment frequency is place on the right-hand side of the table.

Farm	Flock	Season	Flock size	Fattening period	TF	Farm	Median	Mean	IQR	STD
113	1	Winter	30,600	39	61.0	113	23	30.2	7	17.5
113	2	Winter	30,000	36	20.0					
113	3	Summer	30,000	28	23.0					
113	4	Summer	29,500	56	20.0					
113	5	Winter	30,000	35	27.0					
114	1	Winter	67,000	45	39.0	114	32	31.3	12	8.3
114	2	Winter	63,900	39	39.0					
114	3	Summer	65,000	28	32.0					
114	4	Summer	63,000	57	27.0					
114	5	Winter	66,000	35	19.6					
115	1	Winter	52,000	40	29.0	115	29	27.6	10	10.2
115	2	Winter	52,000	36	41.0					
115	3	Summer	52,000	26	14.0					
115	4	Summer	52,000	58	32.0					
115	5	Winter	52,000	33	22.0					
116	1	Winter	26,000	42	29.0	116	24	26.2	7	5.8
116	2	Winter	27,000	37	24.0					
116	3	Summer	25,000	38	35.0					
116	4	Summer	24,500	31	22.0					
116	5	Summer	24,000	43	21.0					

**Figure 1:**
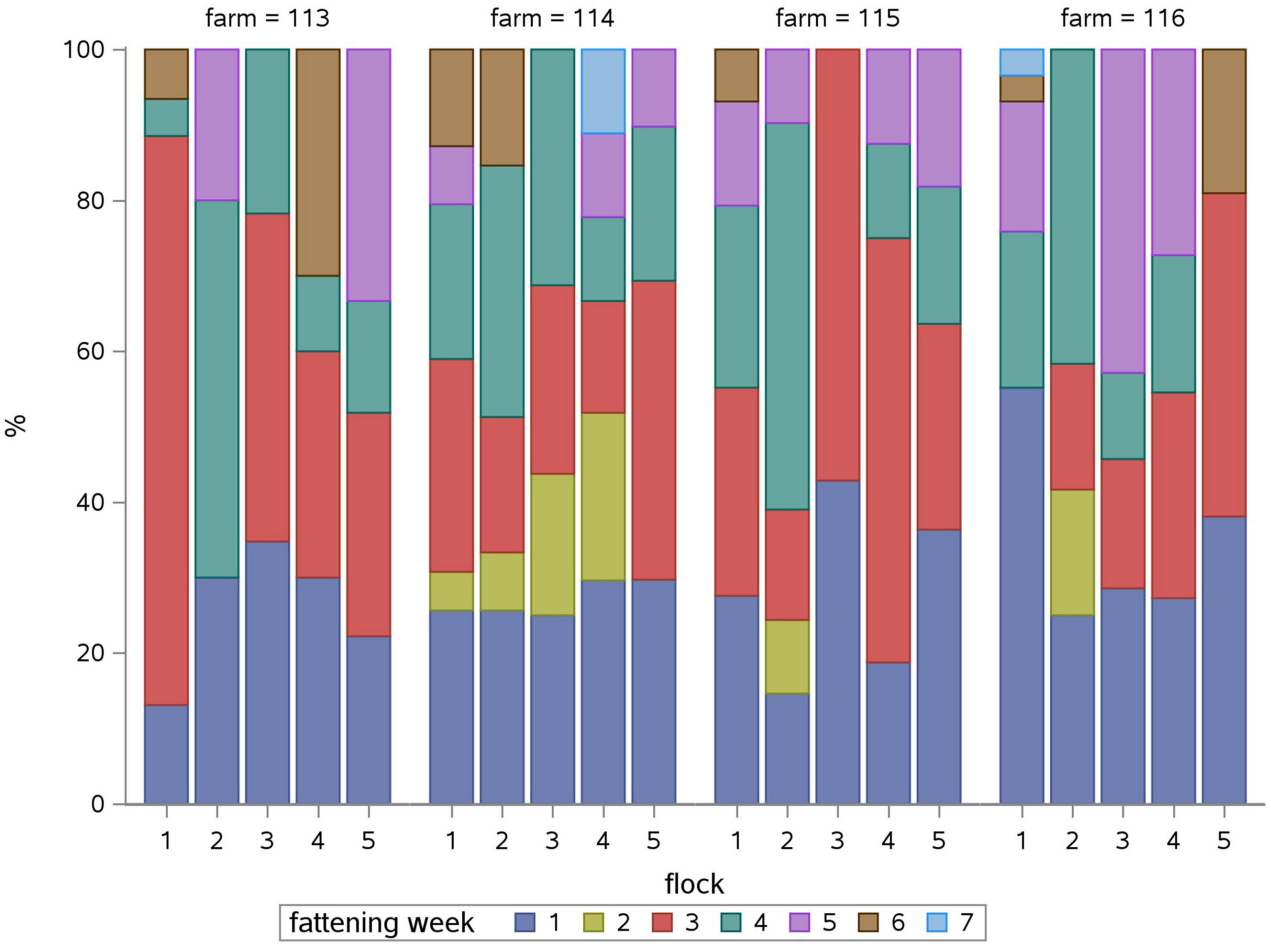
Relative treatment frequency (TF) of each antimicrobial class in percent (%) for each flock (1 to 5) for each farm (113, 114, 115, 116). The relative TF shows, what percentage of the overall flock TF can be attributed to the use of each antimicrobial class.

Overall, polymyxins (27.2%), fluoroquinolones (20.4%), macrolides (17.1) and tetracyclines (15.9%) accounted for the highest proportion of the TF. Figure 1 shows the relative TF for the different antimicrobial classes for each flock in the study period: Some antimicrobial classes were used in all flocks in all farms throughout the year like polymyxins (for growth promotion and *E. coli* infections), macrolides (for *Mycoplasma* infections), fluoroquinolones (for *E. coli* infections) and tetracyclines (for *Mycoplasma* infections), whereas other classes like aminopenicillins, amphenicoles, or nitrofuran derivates were used sporadically in two to three of the farms for *Salmonella* infections.

**Figure 2:**
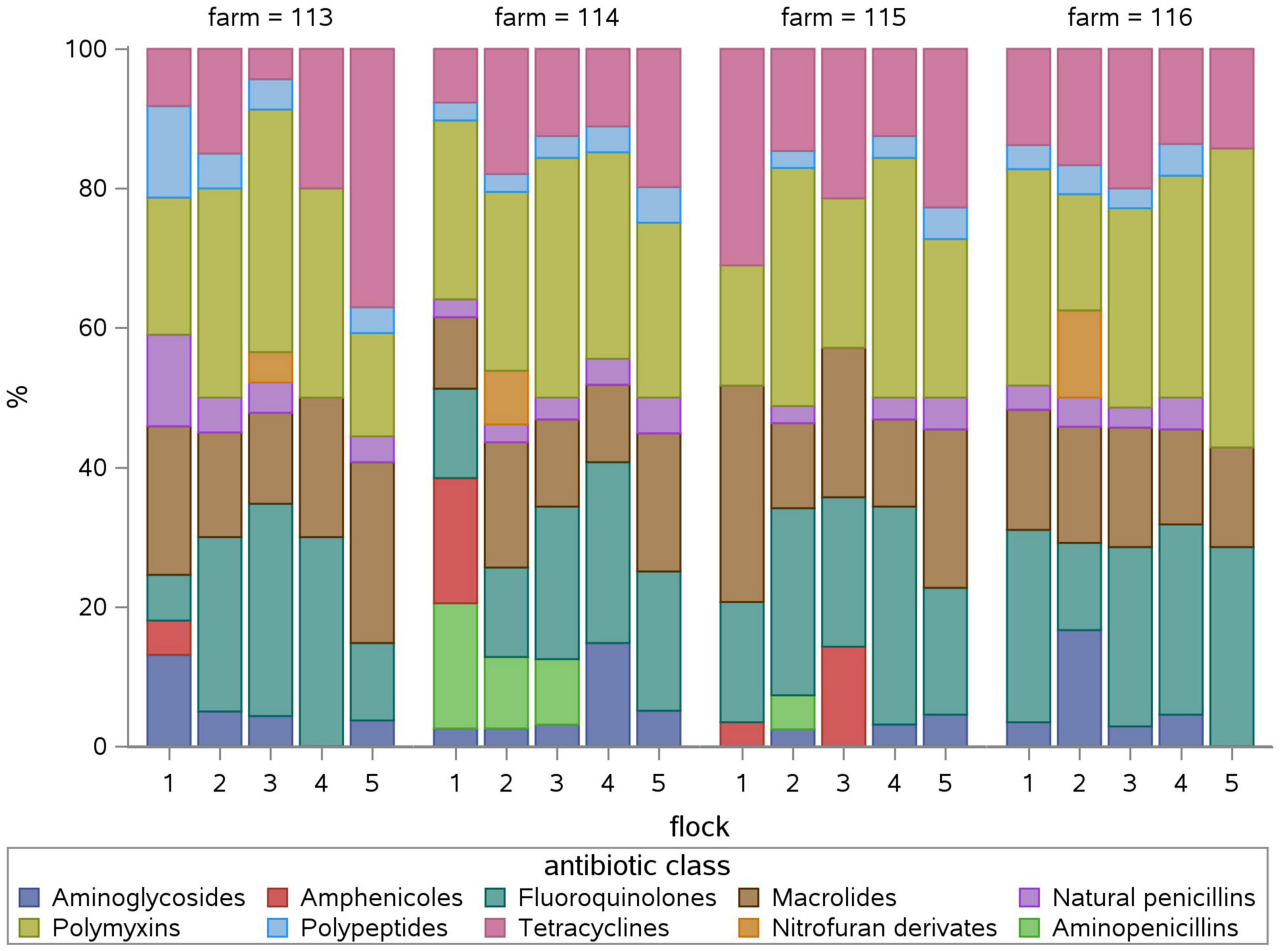
Relative treatment frequency (TF) of each fattening week in percent (%) for each flock (1 to 5) for each farm (113, 114, 115, 116). The relative TF shows, what percentage of the overall flock TF can be attributed to the antimicrobial use in each fattening week.

**Figure 3:**
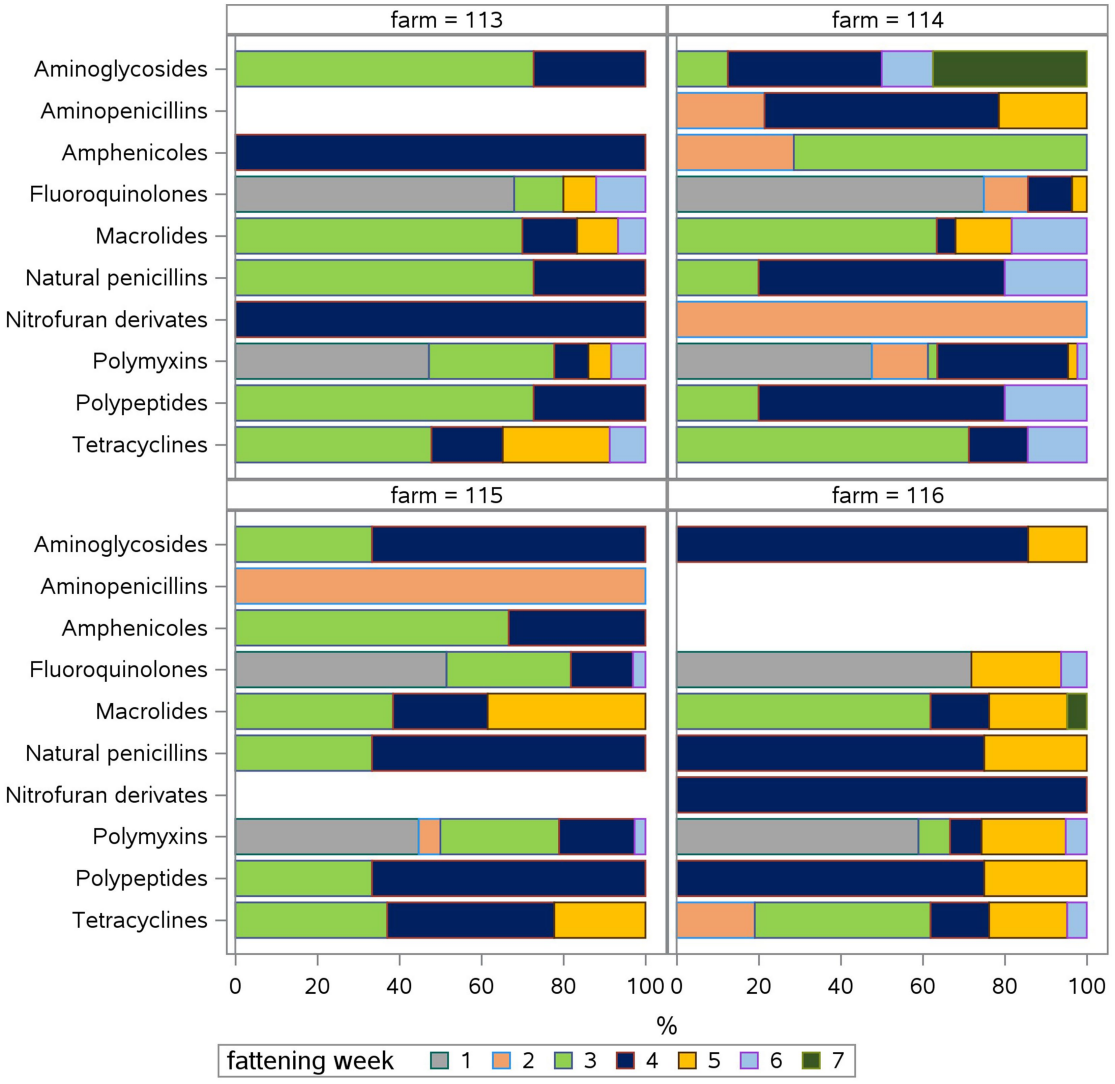
Relative treatment frequency (TF) of each fattening week in percent (%) for each antimicrobial class for each farm (113, 114, 115, 116). The relative TF shows, what percentage of the TF for eacht antimicrobial class can be attributed to the use in each fattening week.

Taking a look at the fattening weeks, all flocks have been treated in the first week of the fattening period and at least once in the third and/or fourth week in all four farms (Figure 2). The median TF was highest in the first (8) and third (7.9) week (Suppl. 2). During the first fattening week, all farms used polymyxins and fluoroquinolones exclusively (Figure 3) due to *E. coli* infections. One farm treated four of five flocks in the second week as well with five different antimicrobial classes due to *E. coli* and *Salmonella* infections. Two farms treated one of five flocks in the second week, one using tetracyclines due to *Mycoplasma* infection, the other using aminopenicillins and polymyxins due to *Salmonella* infection. One farm did not treat any flocks during the second fattening week. From the third week onwards, Growth promotion could be observed as well as treatments due to infections as mentioned above. During the third and fourth fattening week, the greatest variety of antimicrobial classes – seven or more – were used in all farms except one where three antimicrobial classes were used during the third fattening week and seven classes during the fourth. The TF had the highest IQR in the fourth week (Supplementary material 2). Treatments during the fifth and sixth week, again, occurred on all farms with two to six different antimicrobial classes.

### 3.3 Non-parametric tests

Supplementary material 1 shows the group comparisons of overall TF and TF per antibiotic class in regard to season, flock size and duration of the fattening period using median, mean, IQR, standard deviation (Std) as well as wilcoxon two-sample test *p*-values.

The observed differences in overall TF in regard to season, flock size and flock duration were not statistically significant. The lowest *p*-value (*p* = 0.069) was observed for the factor duration of the fattening period. However, a statistically significant difference between summer and winter flocks was seen in the use of fluoroquinolones (*p* = 0.0463) and macrolides (*p* = 0.0325). The TF for Fluoroquinolones was higher in summer (median = 7) than in winter (median = 5) flocks, whereas macrolides were used more often in winter flocks (median = 5) than in summer (median = 4) flocks.

Aminopenicillins were generally used sporadically and not at all in the two smaller farms, which lead to a statistically significant difference regarding flock size as well (*p* = 0.035).

## 4 Discussion

This study investigated the in-farm variation in antimicrobial use of four commercial broiler farms with five flocks each in Faisalabad, Pakistan, for 12 months in 2022 using the international VetCAb-ID database.

The Punjab province accounts for approximately 70% of all poultry farms in Pakistan. In Punjab, relatively modern, economically efficient, environmentally controlled poultry houses typically accommodate 30,000 birds or more (16). According to Punjab Development Statistics (17), there are 12,481 broiler farms in the province, with the highest concentration in Faisalabad (1,382 farms). Therefore, the broiler farms in Faisalabad were considered representative of typical broiler farms in the region. However, the small sample size and regional scope limit the results’ applicability to the entire Pakistani poultry production.

To quantify the use of antimicrobials, the treatment frequency was calculated as described by Kasabova et al. (14) for the overall AMU per flock and fattening week, and for the use of different antimicrobial classes. The evaluation of the in-farm variance showed that the TF for individual flocks exhibits a wide range from 14 to 61. We did not observe any systematic differences in farming practice across the observed farms representing the higher TF values. Importantly, no major disease outbreaks were recorded on the farms during the study period. We could observe, however, that *E. coli* and *Mycoplasma* infections were treated in all flocks, whereas treatments for *Salmonella* infections and growth promotion purposes did not occur in all flocks, which explains a part of the observed differences.

The most common antimicrobials and used in every observed flock were polymyxins (27.2%), fluoroquinolones (20.4%), macrolides (17.1%) and tetracyclines (15.9%). The high use of polymyxins and fluoroquinolones especially raises concerns about AMR risks, because they have been classified as highest-priority critically important antimicrobials by WHO. Polymyxins and fluoroquinolones were found to be the most frequently used antimicrobial classes for prophylactic purposes in previous studies from Pakistan (9, 13). This finding indicates that many farmers rely on preventive antimicrobial use. In our study, the focus is on treating *E. coli* infections at the beginning of the fattening period, and treatments for growth promotion are administered from the third week onwards. This explains the wider range of antimicrobial classes used from the third to the sixth week, especially considering that the drugs used for growth promotion contained active ingredients from four to five antimicrobial classes. Other antimicrobial classes like aminopenicillins or amphenicoles were used in few flocks and not all farms.

While overall TF did not show significant seasonal differences (*p* = 0.57), the median TF was slightly higher in winter than in summer flocks. In a previous study, during the winter season, the AMU data indicated higher use of antimicrobials, particularly for the management of *E. coli* and *Mycoplasma* infections, as well as for prophylactic purposes (18). In this study, the use of macrolides (mainly used for *Mycoplasma* infections) was also statistically significant higher in winter than in summer flocks, but the use of fluoroquinolones was actually higher in summer due to a higher number of *E. coli* and *Salmonella* treatments. We suggest additional data from a larger sample size to clarify on the seasonal influences on infections and treatments.

Larger flock sizes and longer fattening periods showed a clear tendency for higher TF as well but could not be confirmed as statistically significant. We strongly suggest multi-factorial analyses with a larger sample size to explore these factors and their influence on AMU in Pakistani poultry.

Compared to German poultry farms, the median TF in Pakistani poultry farms was more than four times higher. Kasabova et al. (14) showed that no AMU was reported in more than 30% of all broiler flocks whereas in this study in all flocks AMU was observed (19). The use of up to six different antimicrobials at the end of the fattening period was observed. This has also been reported in other low- and middle-income countries (20).

In our study, the median TF (27) at farms was lower compared to a recent study from Punjab, Pakistan where treatment incidence of 75.3 was observed which could likely be due to differences in farm management, and disease burden (21). In addition, the difference of UDD and DDD related measures may also play a role here (14). Both studies showed high use of polymyxins, fluoroquinolones and macrolides. Mahmood et al. found aminoglycosides to be the second most common used antimicrobials. In our study, aminoglycosides were used in all treatments for growth promotion and sporadically for *Salmonella* infections, overall aminoglycosides were used in 18 of 126 treatment in our study. Both studies confirm extensive AMU and use of critically important antimicrobials in Pakistani broiler production.

A comparison with other studies from LMICs was only possible to a limited extent due to differences in systems and metrics used to monitor drug consumption. Therefore, a direct quantitative comparison was not possible. Qualitatively, it was observed that studies from Indonesia, Tanzania and Bangladesh also reported high usage of HPCIAs, such as fluoroquinolones, polymyxins and macrolides, either alone or in combination with other active substances (22–24). Unlike studies from Indonesia and Bangladesh, which did not distinguish between therapeutic use, prophylaxis, and growth promotion, studies from India and Tanzania also reported the use of antimicrobials for growth promotion (22–25). A Nigerian study found that 98% of treatments for laying hens were prophylactic, with antibiotics from six different classes often being used simultaneously (20). Pakistan’s poultry plays crucial role in meeting the protein requirements of the human population which particularly highlights the issue of high AMU for food safety in relation to drug residues and the transmission of AMR. However, weak regulatory oversight and lack of AMU surveillance and benchmarking at farm-level contribute to excessive reliance on antimicrobials. A recent study analysed the implementation challenges of Pakistan’s National Action Plan on AMR in agriculture and food sector and highlighted key barriers such as weak enforcement of AMU regulations, lack of surveillance, and the continued availability of critically important antimicrobials for poultry production (26). Our findings reinforce these concerns, as 79% of the treatments in this study involved highest-priority critically important antimicrobials (HPCIA) drugs. Our data showed HPCIA like polymyxins, fluoroquinolones and macrolides were frequently used which is in consistent with our previous study conducted between 2014 and 2018 (9). Regrettably, this shows that despite the partial implementation of national action plan, the trends in HPCIA use remain unchanged. It is therefore apparent that further efforts are needed to improve education on antibiotic stewardship among poultry farmers and veterinarians, and restrict the access to HPCIA, especially for the use of growth promotion. Implementing documentation practices that allow for evaluations across flocks and farms as presented in this study can help to gather knowledge about the current use and aim to reduce AMU with a benchmarking approach. It needs to be addressed, however, that a system relying only on self-reported data from farmers could lead to an underestimation of the actual use due to farmer’s fear of repercussions.

One limitation of our study is the small sample size, which indeed restricts the precision of the investigation. Possibly influencing factors such as flock size, duration of the fattening period and season could be explored in one-factorial tests only. There were too few observations per subgroup for multi-factorial group comparisons. Confounding factors and interactions could not be assessed. To gain a better understanding of patterns and interactions, a multi-factorial modelling approach considering all mentioned factors as well as flocks as repeated measures or farms as random effects would be advisable with a larger sample size.

Moreover, environmental factors like humidity, stocking density, feed and water quality, biosecurity, vaccination programs, among others, were not analysed in this study. The findings of this study may thus not be directly applicable to the broader poultry industry in Pakistan. However, most of the previous studies in the country were point prevalence surveys (18, 21), whereas this study accounts for antimicrobial use within a well-controlled, closed cohort broiler production unit. In addition, the study controls seasonal trends, flock size and duration and shows the variation from flock to flock within same farms. Hence, this study gives a deeper insight into the variation within a farm and its on-site variation.

To date, there are only a few databases that can be used for direct monitoring of AMU and that have a global reach. Especially in low- and middle-income countries, AMU monitoring at farm level is rare. The database VetCAb-ID provides the infrastructure for this goal, even though a transfer from paper to online based data collection may have to be organized by participating veterinarians and farmers. This study gives an example of how this infrastructure might be used to implement farm level AMU documentation and analysis to learn more about variation between farms and flocks as well as treatments patterns. It could be used to benchmark farms as well. Studies are currently being conducted to investigate the use of antimicrobials in other LMICs using the VetCAb-ID database. The initial results suggest that the database is also suitable for use in other countries. Since no internet access is required to collect antibiotic usage data, the database can also be used to collect data from farms in remote regions. Training videos for project managers and data entry staff are available on the VetCAb-ID homepage. These are also available to the general public.

In the future, the VetCAb-ID database may include environmental factors and thus serve to show immediate consequences of farm management. Overall, the study demonstrates its usefulness for countries where there is no AMU monitoring system in the veterinary sector. Data on AMU in animals can be entered directly into the database and AMU can be described by various subsequent analyses, which again documents the feasibility of monitoring AMU data at the farm level (15).

## 5 Conclusion

This study demonstrates how the documentation of AMU on farms can be systematically organized and evaluated using the global database VetCAb-ID. The findings contribute to a better understanding of the extent and causes of AMU in the poultry industry in Pakistan, revealing frequent use of HPCIA (polymyxins and fluoroquinolones). Such practices pose serious One Health risks by driving antimicrobial resistance and may affect international trade compliance. These findings underscore the urgent need for stronger regulatory enforcement and improved farm-level stewardship. The introduction of a comprehensive monitoring systems at the national level is crucial for making informed policy decisions and effectively reducing the public health risks posed by antimicrobial resistance. Nationwide routine monitoring is essential for developing effective long-term strategies, controlling antibiotic consumption, and protecting human and animal health in the long term.

## Data Availability

The data were collected from farming routine with the understanding that full data would not be transferred to any third party. Therefore, complete data transfer to interested parties is not allowed without an additional formal contract. Data are available to qualified researchers who sign a contract with the project partners. This contract will include guarantees of the obligation to maintain data confidentiality in accordance with the provisions of the European General Data Protection Regulation, its supporting rules in Germany and in Pakistan. Currently, there is no data access committee or another body that could be contacted for the data. However, for this purpose, a committee will be formed. This future committee will consist of members of the University of Veterinary Medicine Hannover and the University of Agriculture, Faisalabad. Interested cooperative partners who are able to sign a contract as described above may contact the corresponding authors.

## Supplementary material

SUPPLEMENTARY FIGURE 1 Treatment frequency by fattening week. Boxplot distribution of the treatment frequency (TF) of all flocks for each fattening week (1–7). The circle inside the box or whiskers represents the mean value, the vertical line inside the box represents the median. Circles outside the whiskers represent outlier values.
