# Supplementary figures and images for: A report of the in-farm variation of antimicrobial use in commercial broiler production in Pakistan using an international monitoring system based on treatment frequency
Source: Front Vet Sci. 2025 Nov 6;12:1650299. doi: 10.3389/fvets.2025.1650299 (PMC12631274; doi:10.3389/fvets.2025.1650299)

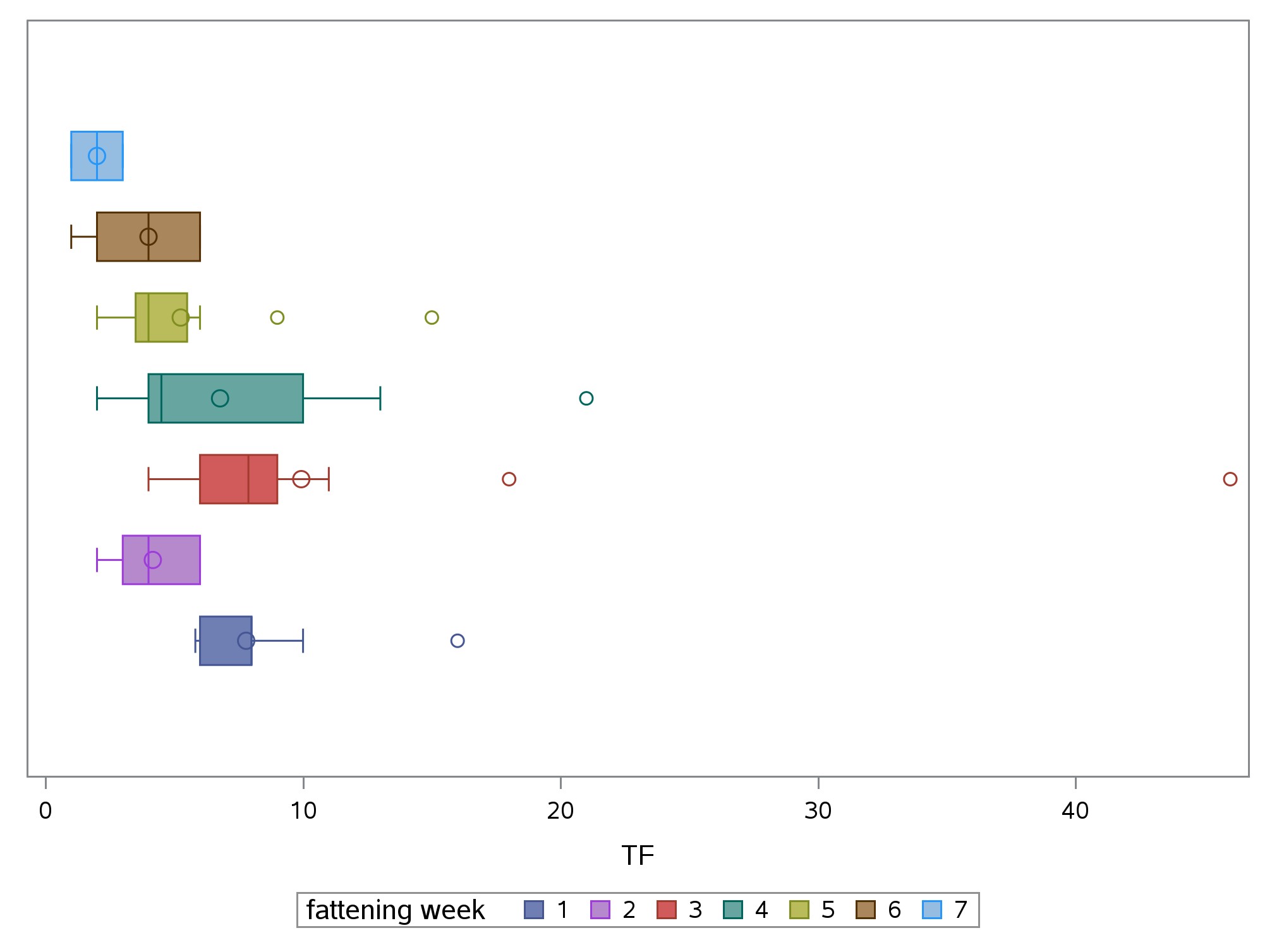

Supplement: SUPPLEMENTARY FIGURE 1 — Treatment frequency by fattening week. Boxplot distribution of the treatment frequency (TF) of all flocks for each fattening week (1–7). The circle inside the box or whiskers represents the mean value, the vertical line inside the box represents the median. Circles outside the whiskers represent outlier values. [file Image_1.jpeg]
