# Supplementary material for: A report of the in-farm variation of antimicrobial use in commercial broiler production in Pakistan using an international monitoring system based on treatment frequency
Source: Front Vet Sci. 2025 Nov 6;12:1650299. doi: 10.3389/fvets.2025.1650299 (PMC12631274; doi:10.3389/fvets.2025.1650299)
Supplement: Supplementary file 2 [file Table_1.docx]

Supplement 1: Comparison of overall TF and TF per antibiotic class in regard to season (summer, winter), flock size (according to the number of day-old chicks placed at the farm) and duration of the fattening period (in days). Median, mean inter quartile range (IQR), standard deviation (Std) and p-values from one-factorial, non-parametric (Wilcoxon two-sample) tests are presented.

| **Antimicrobial class** | **season** | **Median** | **IQR^*^** | **Mean** | **Std** | **p value^**^** |
| --- | --- | --- | --- | --- | --- | --- |
| **total** | summer | 24.00 | 11.00 | 26.45 | 7.81 | 0.5679 |
|  | winter | 29.00 | 17.00 | 31.73 | 13.13 |  |
| **Aminoglycosides** | summer | 1.00 | 1.00 | 1.27 | 1.42 | 0.6866 |
|  | winter | 1.00 | 0.00 | 1.67 | 2.40 |  |
| **Aminopenicillins** | summer | 0.00 | 0.00 | 0.45 | 1.04 | 0.7037 |
|  | winter | 0.00 | 0.00 | 1.22 | 2.54 |  |
| **Amphenicoles** | summer | 0.00 | 0.00 | 0.18 | 0.60 | 0.1922 |
|  | winter | 0.00 | 0.00 | 1.22 | 2.39 |  |
| **Fluoroquinolones** | summer | 7.00 | 3.00 | 6.82 | 2.52 | 0.0463 |
|  | winter | 5.00 | 1.00 | 4.77 | 1.40 |  |
| **Tetracyclines** | summer | 4.00 | 1.00 | 3.82 | 1.60 | 0.1508 |
|  | winter | 5.00 | 3.12 | 5.54 | 2.56 |  |
| **Macrolides** | summer | 4.00 | 1.00 | 3.82 | 0.98 | 0.0325 |
|  | winter | 5.00 | 3.00 | 6.32 | 3.13 |  |
| **Natural Penicillins** | summer | 1.00 | 1.00 | 0.73 | 0.47 | 0.2478 |
|  | winter | 1.00 | 0.00 | 1.67 | 2.40 |  |
| **Polymyxins** | summer | 8.00 | 5.00 | 8.27 | 3.23 | 0.5674 |
|  | winter | 6.00 | 5.00 | 7.32 | 2.92 |  |
| **Nitrofuran derivates** | summer | 0.00 | 0.00 | 0.36 | 0.92 | 0.7597 |
|  | winter | 0.00 | 0.00 | 0.33 | 1.00 |  |
| **Polypeptides** | summer | 1.00 | 1.00 | 0.73 | 0.47 | 0.2478 |
|  | winter | 1.00 | 0.00 | 1.67 | 2.40 |  |
| **Antimicrobial class** | **Flock size (number of day-old chicks)** | **Median** | **IQR^*^** | **Mean** | **Std** | **p value^**^** |
| **total** | 24,000 to 30,600 | 23.50 | 8.00 | 28.20 | 12.44 | 0.4487 |
|  | 52,000 to 67,000 | 30.50 | 17.00 | 29.46 | 8.99 |  |
| **Aminoglycosides** | 24,000 to 30,600 | 1.00 | 0.00 | 1.80 | 2.44 | 0.7212 |
|  | 52,000 to 67,000 | 1.00 | 0.00 | 1.10 | 1.10 |  |
| **Aminopenicillins** | 24,000 to 30,600 | 0.00 | 0.00 | 0.00 | 0.00 | 0.0350 |
|  | 52,000 to 67,000 | 0.00 | 3.00 | 1.60 | 2.41 |  |
| **Amphenicoles** | 24,000 to 30,600 | 0.00 | 0.00 | 0.30 | 0.95 | 0.3305 |
|  | 52,000 to 67,000 | 0.00 | 1.00 | 1.00 | 2.21 |  |
| **Fluoroquinolones** | 24,000 to 30,600 | 6.00 | 3.00 | 5.70 | 2.00 | 0.9696 |
|  | 52,000 to 67,000 | 5.00 | 3.00 | 6.09 | 2.65 |  |
| **Tetracyclines** | 24,000 to 30,600 | 4.00 | 2.00 | 4.40 | 2.50 | 0.6992 |
|  | 52,000 to 67,000 | 4.00 | 3.00 | 4.79 | 1.99 |  |
| **Macrolides** | 24,000 to 30,600 | 4.00 | 3.00 | 5.10 | 3.11 | 0.8165 |
|  | 52,000 to 67,000 | 4.00 | 1.12 | 4.79 | 1.88 |  |
| **Natural Penicillins** | 24,000 to 30,600 | 1.00 | 0.00 | 1.50 | 2.32 | 0.7263 |
|  | 52,000 to 67,000 | 1.00 | 0.00 | 0.80 | 0.42 |  |
| **Polymyxins** | 24,000 to 30,600 | 7.50 | 3.00 | 7.50 | 2.59 | 0.6489 |
|  | 52,000 to 67,000 | 9.00 | 6.00 | 8.19 | 3.56 |  |
| **Nitrofuran derivates** | 24,000 to 30,600 | 0.00 | 0.00 | 0.40 | 0.97 | 0.6264 |
|  | 52,000 to 67,000 | 0.00 | 0.00 | 0.30 | 0.95 |  |
| **Polypeptides** | 24,000 to 30,600 | 1.00 | 0.00 | 1.50 | 2.32 | 0.7263 |
|  | 52,000 to 67,000 | 1.00 | 0.00 | 0.80 | 0.42 |  |
| **Antimicrobial class** | **Flock duration (days)** | **Median** | **IQR^*^** | **Mean** | **Std** | **p value^**^** |
| **total** | 26 to 37 | 22.50 | 7.00 | 24.46 | 7.49 | 0.0690 |
|  | 38 to 58 | 30.50 | 12.00 | 33.20 | 11.75 |  |
| **Aminoglycosides** | 26 to 37 | 1.00 | 0.00 | 1.20 | 1.03 | 0.7549 |
|  | 38 to 58 | 1.00 | 1.00 | 1.70 | 2.50 |  |
| **Aminopenicillins** | 26 to 37 | 0.00 | 0.00 | 0.50 | 1.08 | 0.8712 |
|  | 38 to 58 | 0.00 | 0.00 | 1.10 | 2.42 |  |
| **Amphenicoles** | 26 to 37 | 0.00 | 0.00 | 0.20 | 0.63 | 0.2796 |
|  | 38 to 58 | 0.00 | 1.00 | 1.10 | 2.28 |  |
| **Fluoroquinolones** | 26 to 37 | 4.50 | 4.00 | 5.29 | 2.55 | 0.1583 |
|  | 38 to 58 | 6.00 | 3.00 | 6.50 | 1.96 |  |
| **Tetracyclines** | 26 to 37 | 3.94 | 2.00 | 4.29 | 2.41 | 0.4629 |
|  | 38 to 58 | 4.00 | 4.00 | 4.90 | 2.08 |  |
| **Macrolides** | 26 to 37 | 3.94 | 2.00 | 4.09 | 1.29 | 0.1638 |
|  | 38 to 58 | 4.50 | 3.00 | 5.80 | 3.16 |  |
| **Natural Penicillins** | 26 to 37 | 1.00 | 0.00 | 0.90 | 0.32 | 0.6171 |
|  | 38 to 58 | 1.00 | 1.00 | 1.40 | 2.37 |  |
| **Polymyxins** | 26 to 37 | 5.50 | 4.00 | 6.69 | 3.47 | 0.0630 |
|  | 38 to 58 | 9.50 | 2.00 | 9.00 | 2.16 |  |
| **Nitrofuran derivates** | 26 to 37 | 0.00 | 0.00 | 0.40 | 0.97 | 0.6264 |
|  | 38 to 58 | 0.00 | 0.00 | 0.30 | 0.95 |  |
| **Polypeptides** | 26 to 37 | 1.00 | 0.00 | 0.90 | 0.32 | 0.6171 |
|  | 38 to 58 | 1.00 | 1.00 | 1.40 | 2.37 |  |
